# Recent trend in gastric cancer treatment in the USA

**DOI:** 10.20517/2394-4722.2017.74

**Published:** 2018-04-26

**Authors:** Kazuto Harada, Hideo Baba, Jaffer A. Ajani

**Affiliations:** 1 Department of Gastrointestinal Medical Oncology, University of Texas M. D. Anderson Cancer Center, Houston, TX 77030, USA.; 2 Department of Gastroenterological Surgery, Graduate School of Medical Science, Kumamoto University, Kumamoto 860-8556, Japan.

**Keywords:** Gastric adenocarcinoma, chemotherapy, chemoradiation, preoperative treatment

## Abstract

Gastric adenocarcinoma (GAC) is estimated as the fifteenth most common cancer in the USA. Incidence rate has been gradually decreasing, but prognosis remains dismal. For patients with locally advanced GAC (stage > T1B and < T4B), multimodality therapies, such as surgery, chemotherapy, and radiation therapy, are needed. Perioperative chemotherapy or postoperative chemoradiation/chemotherapy is recommended. For metastatic GAC patients, combination of two cytotoxics (platinum compound and fluoropyrimidine) has become a common place in the USA, and when HER2 is positive, trastuzumab is added. When GAC progresses after the first line therapy, additional biomarkers (microsatellite instability and programmed death ligand 1) should be tested so that checkpoint inhibitors can be used. Overall, the options for advanced GAC patients are limited and more research is needed.

## EPIDEMIOLOGY IN THE USA

Gastric adenocarcinoma (GAC) is estimated as the fifteenth most common cancer in the USA; 28,000 new cases are estimated in a year, which is 1.7% of all new cancer cases^[1]^. Incidence rate has been gradually decreasing; number of new cases per 100,000 people is 11.7 in 1975, 9.3 in 1990, 8.1 in 2000, and 6.6 in 2014^[1]^. In total 10,960 deaths are estimated in a year, which is 1.8% of all cancer death^[1]^. The 5-year survival rate of GAC in the USA is 30.6%; 53% GAC are localized at diagnosis, and the 5-year survival rate of localized GAC (no lymph node involvement) and regional GAC (regional lymph node involvement) is 67.2% and 30.7%, respectively^[1]^; 35% GAC are diagnosed as metastatic disease and have a poor outcome^[1]^.

Location of GAC had dramatically changed in the USA. Most of GAC originate from the proximal lesser curvature, cardia, and the gastroesophageal junction^[2]^. This location trend is considered due to environmental risk factors, such as *Helicobacter pylori* infection, smoking, high salt intake, and obesity.

## STANDARD TREATMENT FOR RESECTABLE GAC IN THE USA

Resectable GAC patients with ≥ cT1b can proceed to surgery (in the community setting) or receive preoperative therapy (in the university setting) [Table 1]. If GAC patients directly undergo surgery, postoperative chemoradiation is recommended based on the pathological stage or quality of surgery. Endoscopic resection is performed according to Japanese guideline^[3]^, but early stage (stage I) GAC is rare in the USA.

At our institution, we prefer the strategy of induction chemotherapy followed by chemoradiation and surgery^[4,5]^. This strategy originated at our institution (also, feasible in multi-institutional settings) and has been pursued based on excellent results recently reported^[5]^. Induction chemotherapy consists of 4 doses 5-fluorouracil (5-FU) and oxaliplatin administered every 2 weeks, and chemoradiotherapy consists of 45 Gy in 25 fractions with concurrent 5-FU/capecitabine with or without another cytotoxic like a platinum compound or taxane (when gastroesophageal junction is involved). After 6-8 weeks from the end of chemoradiation, a D2 dissection is attempted.

### Postoperative chemoradaiation

SWOG 908/INT-0116, which started in 1991, is one of the most cited trials showing the survival benefit of postoperative chemoradiation for resected GAC in the USA^[6,7]^. In this trial, a total of 556 patients who underwent R0 resection were randomly assigned to surgery alone or surgery plus postoperative chemoradiotherapy (bolus 5-FU and leucovorin with 45 Gy radiotherapy). Compared with surgery alone group, postoperative chemoradiotherapy group showed better overall survival (OS) and relapse-free survival (RFS); the hazard ratio (HR) for OS is 1.32 [95% confidence interval (CI) 1.10–1.60; *P* = 0.0046], and the HR for RFS is 1.51 (95% CI 1.25–1.83; *P* < 0.001). Both overall relapse and locoregional relapse were decreased in postoperative chemoradiotherapy group^[6,7]^. According to these results, postoperative chemoradition therapy became the standard treatment. It is appropriate only for those patients who undergo suboptimal surgery and do not received preoperative chemotherapy.

INT 0116 had some inherent drawbacks since surgical method was not part of the protocol. Thus, in the INT-0116 trial, D0, D1, and D2 lymph node dissections underwent in 54%, 36%, and 10% patients, respectively. Therefore, the efficacy of postoperative chemoradiation after D2 resection remains unclear. The ARTIST (Adjuvant Chemoradiation Therapy in Stomach Cancer) trial in Korea compared postoperative treatment with capecitabine plus cisplatin (XP) and XP plus radiation after curative resection with D2 lymph node dissection^[8]^. This trial showed that the estimated 3-year disease free survival rates were 78.2% in the chemoradiation group and 74.2% in XP alone group (*P* = 0.862), suggesting the addition of radiation to adjuvant XP did not significantly reduce recurrence after D2 dissection^[8]^. Additionally, the randomized phase III CRITICS-study assessed perioperative chemo *vs*. postoperative chemoradiation after preoperative chemotherapy. Patients had D1+ dissection with gastrectomy in this trial. In total 788 patients were randomized into chemotherapy group (*n* = 393) and chemoradiation group (*n* = 395), and the 5-year survival is 41.3% for chemotherapy group and 40.9% for chemoradiation group (*P* = 0.99)^[9]^. These results suggest that postoperative chemoradiation is not useful if optimal or near-optimal surgery is performed.

Several chemotherapy regimens before and after chemoradiation were evaluated^[10–12]^. For instance, Korean study evaluated 5-FU plus cisplatin (FP) before and after concurrent radiotherapy with capecitabine, and this regimen was well tolerated^[10]^. Epirubicin, cisplatin, and 5-FU (ECF) before and after concurrent radiotherapy was assessed, and this regimen was feasible, but did not improve survival^[11,12]^.

### Perioperative chemotherapy

Trials evaluating perioperative chemotherapy were held in Europe and its results have impacted NCCN Guideline as category 1 evidence. MAGIC trial showed an advantage in OS but control and experimental arms performed poorly^[13]^. The NCCN guidelines have not downgraded ECF based on toxicity issues and poor efficacy^[13]^. FNCLCC/FFCD trial randomly assigned 224 patients into the 2 groups: 113 to surgery plus perioperative chemotherapy (2 or 3 preoperative and 3 or 4 postoperative cycles of FP) and 111 to surgery alone^[14]^. Compared with the surgery alone group, the perioperative chemotherapy group had a favorable overall survival (5-year rate, 38% *vs*. 24%; HR 0.69; 95% CI 0.50–0.95; *P* = 0.02) and significantly increased the R0 resection rate (84% *vs*. 73%; *P* = 0.04), but 75% of patients in this trial had esophageal adenocarcinoma^[14]^. Recently, MRC-OEO5 trial compared two perioperative chemotherapy regimen, 2 cycles FP and 4 cycles ECF/ECX (epirubicin, cisplatin and capecitabine)^[15]^. This study showed no OS benefit for ECF/ECX compared with FP (3-year rate, 42% *vs*. 39%; HR 0.92; 95% CI 0.79–1.08; *P* = 0.30), suggesting that addition of epirubicin and longer duration does not provide any advantage. However, this trial predominantly included patients with lower esophageal and junctional (types I and II) adenocarcinoma, not GAC.

The FLOT4 trial, which is multicenter, randomized, and phase 3 trial, compared perioperative chemotherapy with docetaxel, oxaliplatin, and fluorouracil/leucovorin (FLOT) and ECF/ECX^[16,17]^. Of 716 patients, 360 patients is assigned into ECF/ECX group and 356 patients assigned into FLOT group, and FLOT improved median progression-free survival (PFS) (30 months *vs*. 18 months; HR 0.75; *P* = 0.001) and median OS (50 months *vs*. 35 months; HR 0.77; *P* = 0.012) compared with ECF/ECX. Fifty percent of patients in FLOT group completed the planned postoperative treatments, while 37% of patients in ECF/ECX completed. Perioperative complications were similar across the 2 groups^[16,17]^. However, the FLOT regimen resulted in considerable toxicity and mortality. Some of the follow up is too early. FLOT could be recommended to only occasional fit patient for perioperative chemotherapy and we don’t recommend it for regular use.

### Preoperative chemoradiation

Preoperative chemoradiation for GAC is not the standard of care in the USA but it is a developing strategy. The strategy has several advantages. Firstly, radiation field is planned more accurately because primary is in place. Postoperative radiation fields were redesigned in about 35% patients in the INT-0116 trial^[6,7]^. Secondly, preoperative chemoradiation increases R0 resection, resulting in low local relapses rate^[5]^. Finally, preoperative chemoradiation might reduce peritoneal dissemination during surgery, however this is debatable.

A multi-institutional trial, where patients received 2 cycles of FP followed by 45 Gy of radiation concurrent with 5-FU, demonstrated that R0 resection rate was 70% and pathologic complete response (pCR) rate was 30^%[18]^. Patients who achieved a good pathological response (< 10% residual carcinoma in the primary) had a significantly longer OS than those who did not (63.9 months *vs*. 12.6 months; *P* = 0.03)^[18]^. In another trial, paclitaxel-based induction chemotherapy and chemoradiotherapy were also assessed. This trial demonstrated that pCR rate was 20%, and over 36 months median survival had been estimated^[19]^. In these trials, laparoscopic staging and endoscopic ultrasonography were used for initial staging. Moreover, surgery was a part of sequential treatment strategy and thus was required to be high quality, such as D2 dissection. Therefore, this strategy was considered to be limited in some specialized institutions. The RTOG 9904 assessed quality, survival, and safety of this strategy with 20 institutions and demonstrated its feasibility. In this trial, the pCR and R0 resection rates were 26% and 77%, respectively. A D2 dissection was performed in 50% of patients^[20]^.

Phase III trials to assess the value of preoperative chemoradiation in GAC, TOPGEAR trial, is currently evaluating the efficacy of adding preoperative chemoradiation to perioperative ECF (MAGIC trial regimen)^[21]^. The CRITICS-II trial started to assess the optimal preoperative regimen by comparing three arms; preoperative chemotherapy followed by surgery, preoperative chemotherapy and subsequent chemoradiation followed by surgery, and preoperative chemoradiation followed by surgery (NCT02931890). Results of these trials are forthcoming.

## STANDARD TREATMENT FOR METASTATIC GAC IN THE USA

### First line therapy

The recommended first-line therapy for patients with good performance status is a 2-drug combination of oxaliplatin plus 5-FU or capecitabine [Table 2]. Trastuzumab is added to the first line cytotoxic therapy in patients with HER2 positive GAC based on the ToGA trial^[22]^. Irinotecan in the first line setting did not produce OS advantage and used only for patients who are unable to tolerate platinum-based chemotherapy^[23–25]^. Three-drug combination of docetaxel, cisplatin, and intravenous 5-FU (DCF) or its modification is used by some but it is discouraged for two reasons: (1) it is toxic and provides marginal OS advantage and (2) it is better to avoid a taxane in the first line because one would not be able to take advantage of paclitaxel and ramucirumab in the second line. ECF is not recommended anymore in this situation^[26]^.

5-FU alone or in combination with various reagents used to be the key chemotherapeutic agent against metastatic GAC in the USA; FAM (5-FU, doxorubicin, and mitomycin), and FAMTX (methotrexate, 5-FU and adriamycin) used to be standard treatment^[27,28]^. EAP (etoposide, adriamycin, and cisplatin) was temporarily used in the 1990s, but was discontinued due to toxicity^[29]^. A randomized trial showed that ECF was better than FAMTX, however remained controversial^[30,31]^.

5-FU-based and cisplatin-based combinations were considered as an acceptable standard therapy according to trial in Asia^[32]^. Then, capecitabine, which is an oral fluoropyrimidine, and oxaliplatin, which is third-generation diaminocyclohexane platinum compound, were developed. A phase III in Germany showed that the combination of fluorouracil, leucovorin, and oxaliplatin improved median PFS compared with fluorouracil, leucovorin, and cisplatin (5.8 months *vs*. 3.9 months), but not siginificant^[33]^. The REAL-2 trial demonstrated possible replacement of 5-FU into capecitabine or cisplatin into oxaliplatin^[34]^. These results have led to trend toward preference of capecitabine plus cisplatin or capecitabine plus oxaliplatin in the USA.

S-1, which is oral fluoropyrimidine preferred in Japan, was reported to be similarly effective for survival with a better toxicity compared with infusional fluorouracil in West^[35,36]^. However, dose of S-1 administered each time in West (25 mg/m^2^) is lower than that in Asia (40–60 mg/body)^[37]^. Thus, more evidence is needed to get acceptance for S-1 in the USA.

DCF was evaluated in a randomized study, V-325 in 2006^[38,39]^. It showed that median OS of DCF was significantly longer than CF (9.2 months *vs*. 8.6 months; *P* = 0.02), but DCF produced more toxicity^[38,39]^. Several modified DCF regimens demonstrated the efficacy and the safety^[40–42]^. Thus, the original DCF is not recommended, and modified DCF is still one of the option in specific cases.

### Second/third line therapy

For second line therapy, ramucirumab (an anti-VEGFR2 monoclonal antibody) is the only molecular-targeted drug with a confirmed minimal survival benefit in a global phase 3 trial. The REGARD trial compared ramucirumab and placebo, and showed that median OS in ramucirumab group was better than that in placebo group (5.2 months *vs*. 3.8 months)^[43]^. The RAINBOW trial compared paclitaxel with and without ramucirumab, and showed that OS in ramucirumab plus paclitaxel was significantly longer than in placebo plus paclitaxel (median 9.6 months *vs*. 7.4 months)^[44]^. Ramucirumab plus paclitaxel is the preferred regimen in the second line setting. Docetaxel, irinotecan and paclitaxel have significantly prolong OS compared to best supportive care, but all these trials were flawed^[45–47]^.

Immune checkpoint blockade has received global attention in recent years^[48–50]^. Keynote-059 assessed efficacy and safety of pembrolizumab, programmed death-1 (PD-1) inhibitor, monotherapy showed that overall response rate (ORR) was 11.2% and median duration of response (DOR) was 8.1 months in all cohort^[51]^. ORR was higher in PD-1 ligand (PD-L1) positive patients than PD-L1 negative patients (15.5% *vs*. 5.5%). Checkmate 032 assessed the combination of two checkpoint inhibitors, nivolumab (PD-1 inhibitor) and ipilimumab (cytotoxic T-lymphocyte-associated protein 4 inhibitor), and showed that ORR for combination therapy in PD-L1 positive patients was 40%, which was higher than nivolumab monotherapy^[52]^. Interestingly, among 7 patients with high microsatellite instability (MSI-H) tumors in Keynote-059, ORR was 57% and the CR rate was 14.3%. Given this result, the FDA has approved pembrolizumab for the treatment of patients with PD-L1 positive GAC who have received 2 or more lines of chemotherapy. Pembro is also approved for MSI-H tumor patients. Therefore, now we have to consider all 3 biomarkers for gastroesophageal adenocarcinoma patients (Her2, PD-L1, and MSI).

## PERSPECTIVE FOR TARGETED THERAPY AND IMMUNOTHERAPY

### Targeted therapies against stem cells

Cancer stem cells possess the capacity to self-renew and to cause the heterogeneous lineages of cancer cells. Several makers and pathways related to gastric cancer stemness have been identified^[53]^. Cancer stem cells are resistant to several chemotherapy, and thus targeting cancer stem cells is a potential therapy to overcome treatment resistance. Two stemness related pathways, Hedgehog and signal transducer and activator of transcription 3 (STAT3) pathway, were assessed in clinical trials so far. Vismodegib, which inhibit Hedgehog signals by binding smoothened (SMO), in combination with FOLFOX was assessed in phase 2, but did not benefit PFS (11.5 months *vs*. 9.3 months; *P* = 0.34)^[54]^. Moreover, BRIGHTER study assessed napabucasin, STAT3 inhibitor, in combination with paclitaxel^[55]^. Although detail result of this trial is not available as of this date, napabucasin did not benefit OS^[55]^. However, these strategies might be effective for tumor with high expression of stem cell markers^[56]^. Further research is expected.

### Immunotherpy

To enhance immune checkpoint blockade therapy, combination with several agents have been assessed. Firstly, DNA methyltransferase inhibitor have been found to upregulate interferon signaling and tumor antigen presentation^[57]^. Therefore, a phase 1/2 study have been evaluating azacitidine in combination with pembrolizumab and epacadostat (NCT02959437). Secondly, because inducible CO-stimulator of T cells (ICOS) activate T cell and stimulate an anti-tumor immune response^[58]^, JTX-2011, an agonist of ICOS, in combination with nivolumab is being assessed (NCT02904226).

## TREATMENT FOR PERITONEAL METASTATIC GAC IN THE USA

Recommended therapy for peritoneal metastasis is systemic chemotherapy or best supportive care^[59]^. Hyperthermic intraperitoneal chemoperfusion (HIPEC) is a potential therapy for peritoneal metastases^[60]^. Our institution performed phase II study which evaluated neoadjuvant laparoscopic HIPEC (mitomycin C 30 mg and cisplatin 200 mg) for GAC patients with peritoneal metastasis^[61]^. Seven patients (37%) had negative peritoneal cytology after HIPEC, and the median OS from the date of diagnosis of metastatic disease was 30.2 months^[61]^. However, performing only HIPEC without systemic therapy might impair control of primary or distant disease. Therefore, further phase II trial of HIPEC (NCT02891447) is ongoing in our institution, and this result is expected.

## SUMMARY

In summary, perioperative chemotherapy or preoperative chemoradiation is recommended for localized advanced GAC. Postoperative chemoradiation is option for GAC patients who undergo surgery without preoperative treatment [Table 3]. Result of trials comparing preoperative chemotherapy to chemoradiation is expected. Treatment strategies for metastatic GAC with HER2 negative is two-drug cytotoxic regimen; a platinum compound and a fluoropyrimidine. For GAC with HER2 positive, trastuzumab should be added. Metastatic GAC should be treated based on global trial [Table 3].

## Figures and Tables

**Table 1.** Summary of NCCN guideline for resectable gastric adenocarcinoma

Stage	Treatment (recommendation category or comments)	Preferred regimen (recommendation category)
cT1a	Surgery	
	Endoscopic resection	
cT1b	Surgery	
cT2 higher	Perioperative chemotherapy (1)	Fluorouracil and cisplatin (1)
	(3 cycle preoperative and 3 cycle postoperative)	Fluoropyrimidine and oxaliplatin (1A)
		Epirubicin, cisplatin/oxaliplatin, and fluoropyrimidine (2B)
	Preoperative chemoradiation (2B)	Paclitaxel and carboplatin(1)
		Fluorouracil and cisplatin (1)
		Fluoropyrimidine and oxaliplatin (1)
	Postoperative chemoradiation (1)	Fluoropyrimidine (1A)
	(for patients without preoperative treatment)	(before and after fluoropyrimidine-based chemoradiation)
	Postoperative chemotherapy (2A)	Capecitabine and oxaliplatin (1)
	(for patients after D2 lymph node dissection)	

**Table 2.** Summary of NCCN guideline for metastatic gastric adenocarcinoma

Line	Preferred regimen (recommendation category)	
First-line therapy	HER2 overexpression	Trastsuzumab combination with fluoropyrimidine and cisplatin (1)
		Trastsuzumab combination with other chemotherapy agents (2b)
	HER2 negative	Fluoropyrimidine and cisplatin (1)
		Fluoropyrimidine and oxaliplatin (2A)
		Paclitaxel withcisplatin or carboplatin (2A)
		Docetaxel with cisplatin (2A)
		Fluoropyrimidine (2A)
		Docetaxel or paclitaxel (2A)
		Fluorouracil and irinotecan (2A)
		DCF modification (2A)
		ECF or ECF modification (2B)
Second-line therapy		Ramucirumab and paclitaxel (1)
		Paclitaxel (1)
		Docetaxel(1)
		Irinotecan (1)
		Ramucirumab (1)
		Fluorouracil and irinotecan (2A)
		Irinotecan and cisplatin (2A)
		Docetaxel and irinotecan (2B)

DCF: docetaxel, cisplatin, and intravenous 5-FU; ECF: epirubicin, cisplatin, and 5-FU; 5-FU: 5-fluorouracil

**Table 3.** Key trials for gastric or gastro-esophageal junction adenocarcinoma

Study	Enrolled number	Treatment	Survival	HR (95% CI)	*P* value	Ref.
Pre or postoperative treatment						
INT-0116	281	Surgery → 5-FU/45 Gy	Median OS: 36 months	1.35 (1.09–1.66)	0.005	[6]
	275	Surgery	Median OS: 27 months			
ARTIST	228	Surgery → XP	3-year DFS: 74%	-	0.86	[8]
	230	Surgery → XP/45 Gy	3-year DFS : 78%			
CRITICS	393	ECC → surgery → ECC	5-year OS: 41%	-	0.99	[9]
	395	ECC → surgery → ECC/45 Gy	5-year OS: 41%			
FNCLCC/	113	CF → surgery (*n* = 113)	5-year rate: 38%	0.69 (0.50–0.95)	0.02	[14]
FFCD	111	Surgery (*n* = 111)	5-year rate: 24%			
MAGIC	250	ECF → surgery → ECF	5-year rate: 36%	0.75 (0.60–0.93)	0.009	[13]
	253	Surgery	5-year rate: 23%			
MRC	446	ECF → surgery	3-year rate: 39%	0.92 (0.79–1.08)	0.30	[15]
OEO-5	451	CF → surgery	3-year rate: 42%			
FLOT4	360	ECF → surgery → ECF	Median OS: 35 months	0.77 (0.63–0.94)	0.012	[17]
	356	FLOT → surgery → FLOT	Median OS: 50 months			
Targeted therapy						
ToGA	298	Trastuzumab + XP	Median OS: 13.8 months	0.74 (0.60–0.91)	0.0046	[22]
	296	Placebo + XP	Median OS: 11.1 months			
REGARD	238	Ramucirumab	Median OS: 5.2 months	0.78 (0.60–0.99)	0.047	[43]
	117	Placebo	Median OS: 3.8 months			
RAINBOW	330	Ramucirumab + paclitaxel	Median OS: 9.6 months	0.81 (0.68–0.96)	0.017	[44]
	335	Placebo + paclitaxel	Median OS: 7.4 months			

OS: overall survival; DFS: disease free survival; HR: hazard ratio; CI: confidence interval; XP: cisplatin and capecitabine; ECC: epirubicin, cisplatin and capecitabine; CF: cisplatin and 5-FU; ECF: epirubicin, cisplatin and 5-FU; FLOT: docetaxel, oxaliplatin, leucovorin, and 5-FU; 5-FU: 5 fluorouracil
